# Supplementary figures and images for: Ground-truth-free deep learning approach for accelerated quantitative parameter mapping with memory efficient learning
Source: PLoS One. 2025 Jun 2;20(6):e0324496. doi: 10.1371/journal.pone.0324496 (PMC12129214; doi:10.1371/journal.pone.0324496)

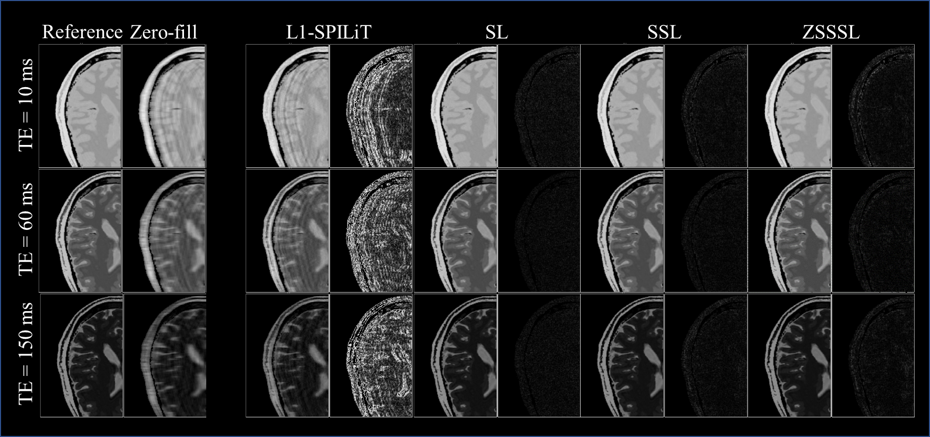

Supplement: S1 Fig — Error images relative to the fully sampled reference image are displayed with x10 scaling. SL: Supervised Learning, SSL: Self-Supervised Learning, ZSSSL: Zero-Shot Self-Supervised Learning. (TIF) [file pone.0324496.s001.tif]

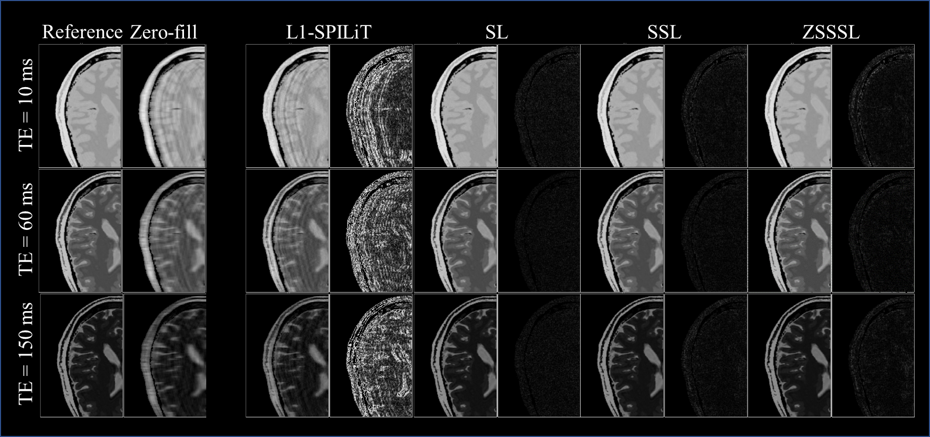

Supplement: S2 Fig — Error images relative to the fully sampled reference image are displayed with x10 scaling. SL: Supervised Learning, SSL: Self-Supervised Learning, ZSSSL: Zero-Shot Self-Supervised Learning. (TIF) [file pone.0324496.s002.tif]

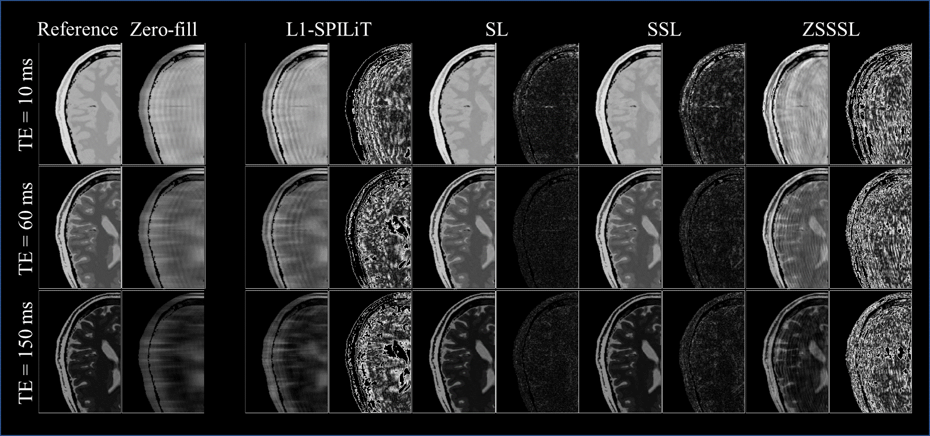

Supplement: S3 Fig — Error images relative to the fully sampled reference image are displayed with x10 scaling. SL: Supervised Learning, SSL: Self-Supervised Learning, ZSSSL: Zero-Shot Self-Supervised Learning. (TIF) [file pone.0324496.s003.tif]

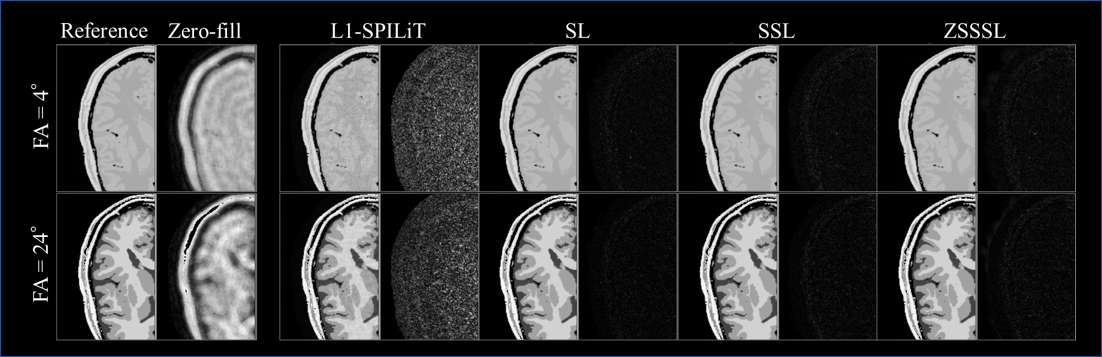

Supplement: S4 Fig — Error images relative to the fully sampled reference image are displayed with x10 scaling. SL: Supervised Learning, SSL: Self-Supervised Learning, ZSSSL: Zero-Shot Self-Supervised Learning. (TIF) [file pone.0324496.s004.tif]

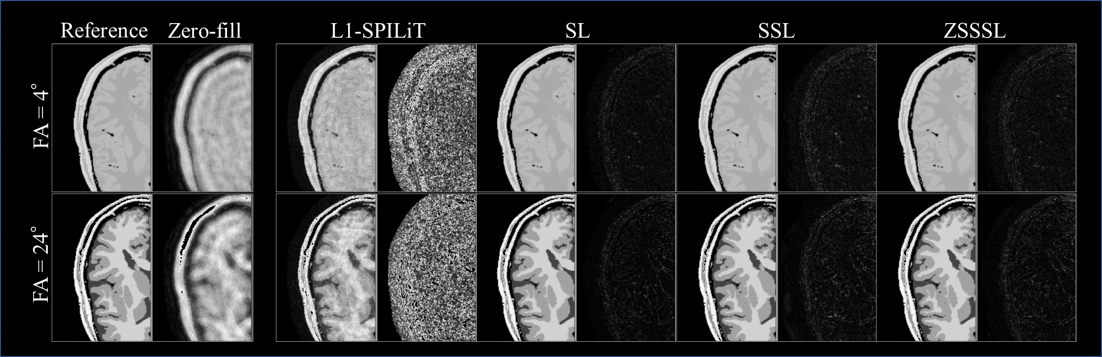

Supplement: S5 Fig — Error images relative to the fully sampled reference image are displayed with x10 scaling. SL: Supervised Learning, SSL: Self-Supervised Learning, ZSSSL: Zero-Shot Self-Supervised Learning. (TIF) [file pone.0324496.s005.tif]

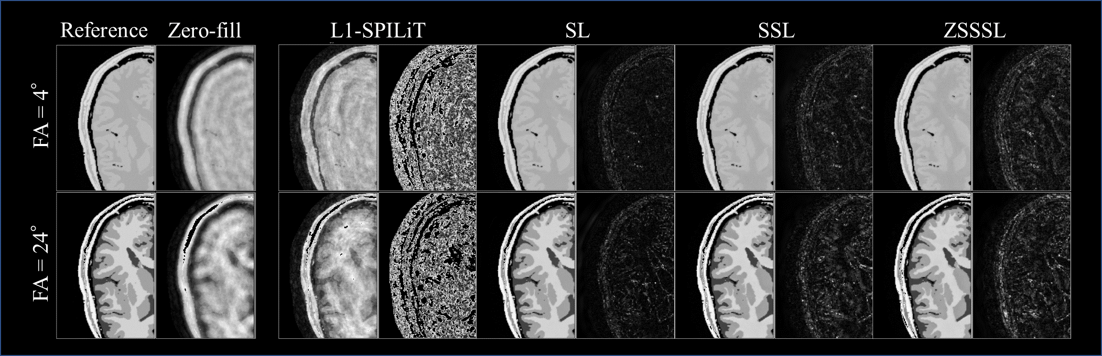

Supplement: S6 Fig — Error images relative to the fully sampled reference image are displayed with x10 scaling. SL: Supervised Learning, SSL: Self-Supervised Learning, ZSSSL: Zero-Shot Self-Supervised Learning. (TIF) [file pone.0324496.s006.tif]

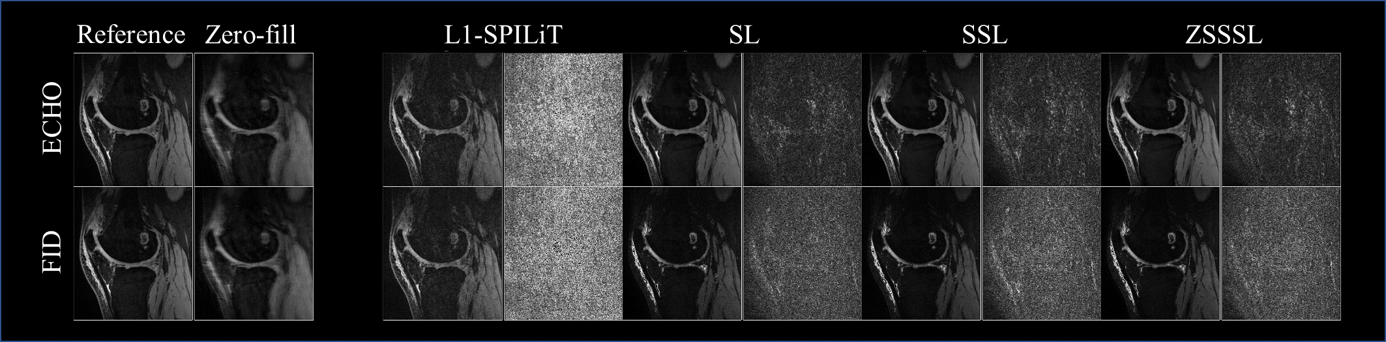

Supplement: S7 Fig — Error images relative to the fully sampled reference image are displayed with x10 scaling. SL: Supervised Learning, SSL: Self-Supervised Learning, ZSSSL: Zero-Shot Self-Supervised Learning. (TIF) [file pone.0324496.s007.tif]

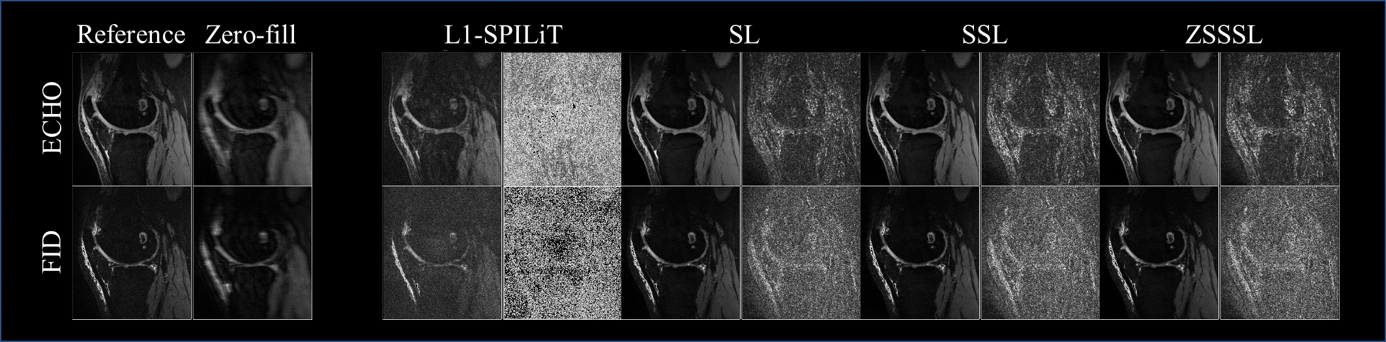

Supplement: S8 Fig — Error images relative to the fully sampled reference image are displayed with x10 scaling. SL: Supervised Learning, SSL: Self-Supervised Learning, ZSSSL: Zero-Shot Self-Supervised Learning. (TIF) [file pone.0324496.s008.tif]

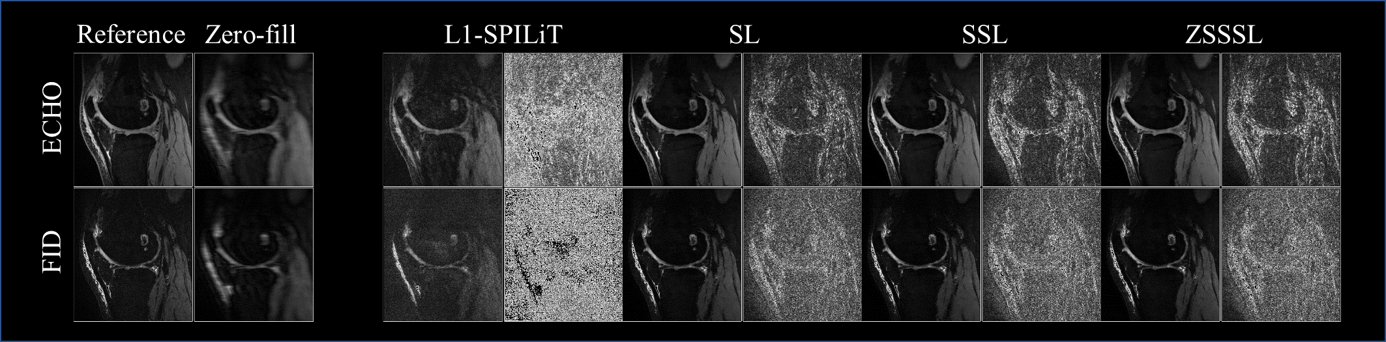

Supplement: S9 Fig — Error images relative to the fully sampled reference image are displayed with x10 scaling. SL: Supervised Learning, SSL: Self-Supervised Learning, ZSSSL: Zero-Shot Self-Supervised Learning. (TIF) [file pone.0324496.s009.tif]
