# Supplementary material for: Ground-truth-free deep learning approach for accelerated quantitative parameter mapping with memory efficient learning
Source: PLoS One. 2025 Jun 2;20(6):e0324496. doi: 10.1371/journal.pone.0324496 (PMC12129214; doi:10.1371/journal.pone.0324496)
Supplement: S1 Data — S1 Table. Quantitative evaluation for contrast images in Experiment 1. The numbers in the table represent SSIM. SL: Supervised Learning, SSL: Self-Supervised Learning, ZSSSL: Zero-Shot Self-Supervised Learning. S2 Table. Quantitative evaluation for contrast images in Experiment 2. The numbers in the table represent SSIM. SL: Supervised Learning, SSL: Self-Supervised Learning, ZSSSL: Zero-Shot Self-Supervised Learning. S3 Table. Quantitative evaluation for contrast images in Experiment 3. The numbers in the table represent SSIM. SL: Supervised Learning, SSL: Self-Supervised Learning, ZSSSL: Zero-Shot Self-Supervised Learning. (PDF) [file pone.0324496.s010.pdf]

S1 Table.

Contrast	Methods	Acceleration factor 4	8	12
TE = 10ms	L1-SPILiT	0.83 ± 0.03	0.78 ± 0.03	0.75 ± 0.02
	SL	**0.99 ± 0.00**	**0.98 ± 0.00**	**0.97 ± 0.00**
	SSL	**0.99 ± 0.00**	0.97 ± 0.00	0.96 ± 0.01
	ZSSSL	**0.99 ± 0.00**	**0.98 ± 0.01**	0.71 ± 0.04
TE = 60 ms	L1-SPILiT	0.86 ± 0.03	0.80 ± 0.03	0.73 ± 0.02
	SL	**0.99 ± 0.00**	**0.99 ± 0.00**	**0.99 ± 0.00**
	SSL	**0.99 ± 0.00**	**0.99 ± 0.00**	**0.99 ± 0.00**
	ZSSSL	**0.99 ± 0.00**	**0.99 ± 0.00**	0.80 ± 0.04
TE = 150 ms	L1-SPILiT	0.82 ± 0.03	0.75 ± 0.03	0.69 ± 0.04
	SL	**0.99 ± 0.00**	**0.99 ± 0.00**	**0.98 ± 0.00**
	SSL	**0.99 ± 0.00**	**0.99 ± 0.00**	**0.98 ± 0.00**
	ZSSSL	**0.99 ± 0.00**	**0.99 ± 0.00**	0.80 ± 0.04

S2 Table.

Contrast	Methods	Acceleration factor 4	8	12
FA = 4°	L1-SPILiT	0.76 ± 0.02	0.61 ± 0.02	0.53 ± 0.02
	SL	**0.99 ± 0.00**	**0.98 ± 0.00**	**0.97 ± 0.00**
	SSL	**0.99 ± 0.00**	0.96 ± 0.00	0.92 ± 0.00
	ZSSSL	0.98 ± 0.01	0.97 ± 0.00	0.94 ± 0.01
FA = 24°	L1-SPILiT	0.91 ± 0.01	0.77 ± 0.01	0.66 ± 0.02
	SL	**1.00 ± 0.00**	**1.00 ± 0.00**	**0.99 ± 0.00**
	SSL	**1.00 ± 0.00**	0.99 ± 0.00	0.98 ± 0.00
	ZSSSL	0.99 ± 0.00	0.99 ± 0.00	0.98 ± 0.00

S3 Table.

Contrast	Methods	Acceleration factor 4	8	12
FID	L1-SPILiT	0.48 ± 0.05	0.35 ± 0.05	0.38 ± 0.05
	SL	0.89 ± 0.01	**0.84 ± 0.02**	**0.81 ± 0.02**
	SSL	**0.90 ± 0.01**	**0.84 ± 0.02**	0.79 ± 0.03
	ZSSSL	0.88 ± 0.01	0.82 ± 0.02	0.80 ± 0.02
ECHO	L1-SPILiT	0.35 ± 0.05	0.23 ± 0.05	0.29 ± 0.05
	SL	**0.85 ± 0.02**	**0.79 ± 0.03**	**0.76 ± 0.03**
	SSL	**0.85 ± 0.02**	**0.79 ± 0.03**	0.74 ± 0.04
	ZSSSL	0.83 ± 0.02	0.76 ± 0.03	0.74 ± 0.03
