# Supplementary material for: Ground-truth-free deep learning approach for accelerated quantitative parameter mapping with memory efficient learning
Source: PLoS One. 2025 Jun 2;20(6):e0324496. doi: 10.1371/journal.pone.0324496 (PMC12129214; doi:10.1371/journal.pone.0324496)
Supplement: S1 File — (DOCX) [file pone.0324496.s011.docx]

# Supplementary Materials

## Metrics

The structural similarity (SSIM) is defined as follows:

$$\begin{aligned} SSIM(x,y)= \frac{\left( 2\mu_{x}\mu_{y}+C_{1} \right)\left( 2\sigma_{xy}+C_{2} \right)}{\left( \mu_{x}^{2}+\mu_{y}^{2}+C_{1} \right)\left( \sigma_{x}^{2}+\sigma_{y}^{2}+C_{2} \right)}\#\left( S1 \right) \end{aligned}$$

Here, $x$ and $y$ represent the ground truth (GT) image and the output image, $\mu_{x}$and $\mu_{y}$ are the mean pixel values of $x$ and $y$, $\sigma_{x}$ and $\sigma_{y}$ are the standard deviations of the pixel values of $x$ and $y$, and $\sigma_{xy}$ is the covariance of the pixel values of $x$ and $y$. The constants $C_{1}={(K_{1}L)}^{2}$ and $C_{2}={(K_{2}L)}^{2}$, where L is the dynamic range of the pixel values, and $K_{1}=0.01$, $K_{2}=0.03$ are constants. A higher SSIM value, closer to 1, indicates better image quality. The normalized Root Mean Squared Error (NRMSE) is defined as follows:

$$\begin{aligned} NRMSE(x,y)=\frac{\left\| x-y \right\|_{2,\Phi}}{\left\| x \right\|_{2,\Phi}}\#\left( S2 \right) \end{aligned}$$

Here, $\left\| \cdot\right\|_{2,\Phi}$ represents the L2 norm over the evaluation region $\Phi$. A lower NRMSE value indicates better image quality. In this study, the evaluation region $\Phi$ was set separately for each experiment.
