# Supplementary material for: Ground-truth-free deep learning approach for accelerated quantitative parameter mapping with memory efficient learning
Source: PLoS One. 2025 Jun 2;20(6):e0324496. doi: 10.1371/journal.pone.0324496 (PMC12129214; doi:10.1371/journal.pone.0324496)
Supplement: Attachment — Submitted filename: reply_to_reviewers.docx [file pone.0324496.s013.docx]

This document (**reply_to_reviewer.docx**) contains the text of the response to the reviewer and the revised text of the manuscript. Here, the referee’s comments were marked in light blue, the responses were in black, and the revisions were recorded using the track changes in Word. The page and line numbers are those in the highlighted version of the revised manuscript (**manuscript_rev_highlighted.docx**).

In addition, to correct grammatical errors and improve overall readability, we used a professional English proofreading service by a native speaker. Please note that these corrections are not individually mentioned in the manuscript.

We have included three newly created figures (**Fig7.tif**, **Fig9.tif**, and **Fig11.tif**) to improve the visual presentation of the quantitative results. As a result of adding these figures, the numbering of the subsequent figures has shifted: the original Fig 7 is now Fig 8, Fig 8 is now Fig 10, and Fig 9 is now Fig 12 in the revised manuscript.

*Reviewer #1: *Reviewer recommendation: Accept with minor revisions.

Detailed review:

[R1-1] Provide an abbreviation glossary and ensure each term is clearly defined when introduced.

Thank you for pointing this out. In response to your comment, we have reviewed the manuscript to ensure that all abbreviations are clearly defined upon their first appearance. Specifically, we have revised the following sentences:

Introduction: page 3, line 2

Compressed sensing (CS)[7], which leverages the inherent sparsity of MR images and parallel imaging (PI)[8–11], which uses multiple receiver coils are also used to accelerate qMRI by reducing the number of data points acquired.

Introduction: page 3, line 6

The DL framework commonly used in image reconstruction is supervised learning (SL), where many fully sampled (FS) data that satisfy the Nyquist condition are used to train the model parameters for reconstruction from undersampled (US) data that do not satisfy the Nyquist condition. Reconstruction models using SL have also been applied to accelerate qMRI.

Material and Methods: page 8, line 22

The US k-space data used for training and testing were generated by retrospective sampling from the FS data under different acceleration factors (AFs), which represent the degree of acceleration relative to the acquisition time of the FS data (e.g., AF = 2 corresponds to a two-fold acceleration).

Additionally, to further improve clarity and address a similar concern raised by Reviewer 2 (please see [R2-3]), we have added a “Background” section. This new section introduces and explains the technical terms in this study, thereby enhancing the overall accessibility of the manuscript.

[R1-2] Include discussion points on how this work might influence clinical workflows and address any potential biases or risks in real-world applications.

Thank you for the comment. We have added a brief discussion on the clinical impact of our method, the need for standardization, and potential real-world biases. The revised text is as follows:

Discussion: page 19, line 20

The GT-free learning method and memory-efficient strategy used in this study are expected to shorten the total examination time of protocols incorporating conventional qMRI and enable the integration of qMRI sequences that were previously challenging due to long scan durations. SSL technology allows for the construction of DL models using only undersampled (US) data, even for qMRI sequences where acquiring fully sampled (FS) data is time-consuming. Additionally, ZSSSL facilitates the application of DL models to emerging qMRI sequences that are not yet widely adopted and lack large-scale training datasets. When incorporated into clinical workflows, these methods are expected to enhance objective diagnosis by utilizing qMRI as an imaging biomarker. However, to ensure the clinical relevance of observed quantitative values, a standardized workflow from image acquisition to quantitative analysis, including diagnosis and treatment planning, must be established. Such standardization will be essential alongside advancements in accelerated qMRI techniques.

[R1-3] The reconstruction time (over 3 hours per volume for ZSSSL) is a significant limitation for clinical use. Expand the discussion of strategies to mitigate this issue, such as transfer learning or parameter reduction, in future work.

Thank you for pointing out this important limitation. We have expanded the Discussion (page 20, line 17) to address potential strategies to reduce reconstruction time, such as transfer learning with SSL-pretrained weights, use of natural image or simulation-based pretraining, and model simplification via Neural Architecture Search. The revised text is as follows:

Material and Methods: page 8, line 22

Although ZSSSL is highly effective because it does not require a training dataset, it has the drawback of lengthy reconstruction time. In this study, the reconstruction time per volume exceeded three hours, which may be acceptable in preclinical settings but is not feasible for clinical use. One potential solution to reduce the reconstruction time is to employ transfer learning (TL) using weights pretrained with SSL. Yaman et al. [37] reported that this TL approach effectively improves performance and reduces reconstruction time to less than one-tenth. Alternatively, TL using weights pretrained with natural images [44,45] or simulation-based training datasets [46] may also be effective. Another approach is to decrease the number of model parameters, which can be achieved through Neural Architecture Search (NAS)[47]. NAS is an automated technique for finding optimal neural network architectures by exploring a predefined search space, potentially reducing the model size while maintaining or improving the performance. Because the network used in this study has not yet undergone sufficient tuning of the hyperparameters such as the number of filters and layers, optimizing the model architecture remains a topic for future work.

[R1-4] Ensure figures and tables are fully interpretable with detailed captions and legible text.

Thank you for your comment. We have revised the figure captions to include more detailed explanations and ensured that all abbreviations are defined. In addition, we have checked label clarity to improve readability. The updated captions can be found in the revised manuscript as follows:

Material and Methods: page 10, line 18

**Fig 2. Architecture of the image reconstruction model used in this study.** $D_{w}$ corresponds to denoising in Equation (12) and DC corresponds to data consistency in Equation (13). The multi-contrast US k-space is the input and the multi-contrast reconstruction k-space is the output. Because MRI images are complex-valued, the input and output of the denoising unit are concatenated in real and imaginary parts in the channel dimension direction. US: Undersampled, CNN: Convolutional neural network.

Material and Methods: page 10, line 30

**Fig 3. Training strategy in this study.** The figure illustrates the training, validation, and testing procedures for supervised learning, self-supervised learning, and zero-shot SSL. Ω is the original US k-space. Θ, Λ, Ξ, and Γ are subsets of the k-spaces. US: Undersampled.

Material and Methods: page 14, line 27

**Fig 4. Simulation procedures for Experiment 1 and Experiment 2.** The blue box represents the simulation process of multi-contrast data. The yellow box illustrates the procedure for generating the fully sampled (FS) and undersampled (US) k-space data and coil sensitivity maps for training and testing in this study. US: Undersampled, FS: Fully sampled, GT: ground truth.

Material and Methods: page 14, line 31

**Fig 5. coil arrangement used to simulate multi-coil signals.** (Left) The red cube represents the field of view (FOV), showing the spatial arrangement of the receiver coils. Different colors indicate the wiring patterns of each coil. (Right) A 2D projection of the coil arrangement. Each coil is modeled as a loop coil with a radius of 100 mm.

Material and Methods: page 19, line 7

**Fig 12. Memory consumption and training time per minibatch in each network parameter settings.** (a) Maximum memory usage as a function of the number of filters, layers, and iterations. The solid blue line represents results with memory-efficient learning (w/ MEL), while the dashed orange line represents results without memory-efficient learning (w/o MEL). (b) Training time per minibatch.

[R1-5] Strengthen the discussion around plans for prospective validation and explain how the method may perform under real-world imaging conditions.

Thank you for the helpful comment. We have expanded the Discussion (page 21, line 22) to clarify the limitations of retrospective design and to describe plans for prospective validation. The revised text is as follows:

Discussion: page 21, line 22

Second, the design of this study was retrospective, and its performance has not been verified using prospective data. In actual scans, imperfect imaging conditions, such as inaccurate sampling patterns due to eddy current effects, may degrade the reconstruction performance. For example, the B0 magnetic field inhomogeneity and eddy currents can cause geometric distortion and signal loss [55]. Therefore, when planning a prospective study, it may be necessary, for example to assess the impact of eddy currents on image distortion and trajectory deviation through spatiotemporal measurement of k-space trajectories using a field camera [56], and to evaluate the distortion itself through phantom study [57]. Validation with prospectively acquired US k-space data is a future issue, particularly in a study design such as the present study, which used only US k-space data.

*Reviewer #2:*

[R2-1] The manuscript includes many technical terms and acronyms (e.g., CS, PI, SL, SSL, ZSSSL, MEL, GT, AF) without always defining them. This could make the text difficult to follow for readers outside the field.

Thank you for the comment. As noted in our response to [R1-1], we have revised the manuscript to define all technical terms and acronyms upon first use. In addition, we have added a Background section to improve clarity, as also explained in our response to [R2-3].

[R2-2] The manuscript is highly technical, and some sections (e.g., network architecture, training strategies) are difficult to follow for readers who are not experts in MRI reconstruction or deep learning.

Thank you for the comment. We have added explanations to improve clarity where possible. For specific revisions addressing this point, please refer to our responses to [R2-3], [R2-4], and [R2-8].

[R2-3] Add a background section to explain key terms, concepts, and MRI sequences. The paper would benefit from the addition of a background section to improve accessibility and readability. Many key concepts such as Self-Supervised Learning (SSL), Zero-Shot Self-Supervised Learning (ZSSSL), Memory Efficient Learning (MEL), and the qMRI sequences (MSME, VFA-SPGR, DESS) are central to the study but are not explained in sufficient detail for readers who may not be familiar with these techniques.

Thank you for the helpful suggestion. To improve accessibility and readability, we have added a new Background section. This section introduces key concepts, technical terms, and MRI sequences used in the study, including Self-Supervised Learning (SSL), Zero-Shot SSL (ZSSSL), and the qMRI sequences (MSME, VFA-SPGR, qDESS). The added section is located at the beginning of the manuscript and provides the necessary context for readers who may not be familiar with MRI reconstruction or deep learning. For a detailed explanation of Memory-Efficient Learning (MEL), please refer to our response to [R2-8].

# **Background**

**Problem formulation**

In this section, we formulize the MRI sampling process. Under multi-coil conditions, the sampling process can be formalized as follows:

$$\begin{aligned} y_{\Omega}=E_{\Omega}x\#\left( 1 \right) \end{aligned}$$

where $\boldsymbol{x}\in\mathbb{R}^{H\times W\times2}$ is the target image, and $y_{\Omega}\in\mathbb{R}^{C\times H\times W\times2}$ represents the US multi-coil k-space data. Ω is the sampling region, C is the number of receiving coils, and H×W is the image matrix size. The encoding operator $\boldsymbol{E}_{\boldsymbol{\Omega}}:\mathbb{R}^{H\times W\times2}\mapsto\mathbb{R}^{C\times H\times W\times2}$ in PI is defined as:

$$\begin{aligned} E_{\Omega}=\left[ \begin{aligned} M_{\Omega}FC_{1} \\ M_{\Omega}FC_{2} \\ \vdots\\ M_{\Omega}FC_{C} \end{aligned} \right]\#\left( 2 \right) \end{aligned}$$

where $\boldsymbol{C}_{\boldsymbol{i}}$ is the sensitivity of the receiving coil, $\boldsymbol{F}$ is the discrete Fourier operator, and $\boldsymbol{M}_{\boldsymbol{\Omega}}$ is the sampling operator that fills unmeasured k-space points (outside $\Omega$) with zeros.

In this study, we extended the encoding operator in Equation (2) to the sampling process for multicontrast data. In multicoil/multicontrast data acquisition, different sampling regions are often employed for each contrast; thus, the multicontrast extension of the encoding operator in Equation (2) is defined as:

$$\begin{aligned} E_{\Omega}=\left[ \begin{aligned} E_{\Omega_{1}} \\ E_{\Omega_{2}} \\ \vdots\\ E_{\Omega_{P}} \end{aligned} \right]\#\left( 3 \right) \end{aligned}$$

Thus, the operator $\boldsymbol{E}_{\boldsymbol{\Omega}}:\mathbb{R}^{P\times C\times H\times W\times2}\mapsto\mathbb{R}^{P\times H\times W\times2}$ is the encoding operator for multi-contrast k-space data, where *P* is the number of input contrast images, and $\Omega_{i}$ is the sampling region in k-space for the i-th contrast. Here, $\Omega=\{\Omega_{1},\Omega_{2},\ldots,\Omega_{P}\}$ represents the sampling regions for all contrasts. Using this operator, the sampling operation for multi-coil / multi-contrast data can be expressed similarly to Equation (1). Henceforth, unless otherwise specified, the encoding operator in this paper refers to the multicontrast extended encoding operator defined in this section.

**Quantitative MRI sequences in this study**

This section provides an overview of the qMRI sequences used in the present study. In this study, MSME, VFA-SPGR, and qDESS were adopted as standard methods for 2D T2, 3D T1, and fast 3D T2 mapping, respectively.

MSME is a pulse sequence used for T2 mapping that combines multi-slice imaging that excites multiple slices in parallel with the spin-echo technique, where a 90-degree RF pulse excites nuclear magnetization and a 180-degree RF pulse refocuses it to generate echo signals. MSME acquires multiple spin echoes at different echo times (TE) from a single excitation, thus enabling efficient T2 mapping. The signal model is expressed as follows:

$$\begin{aligned} S_{i}\left( I_{0},T_{2} \right)=I_{0}e^{-TE_{i}/T_{2}}\#\left( 4 \right) \end{aligned}$$

where $I_{0}$ and $T_{2}$ represent the proton density image and T2 map, respectively. ${TE}_{i}$ represents the echo time of the ith echo. Using the signal model in (4), the T2 map can be estimated using least-squares fitting (LSF).

VFA-SPGR is a widely used method for T1 mapping, particularly for acquiring 3D T1 maps [29]. This method uses SPGR sequences acquired multiple times with multiple FA and constant TR and TE. The signal model for the SPGR sequence is as follows:

$$\begin{aligned} S_{i}\left( I_{0},T_{1} \right)=I_{0}\frac{(1-e^{-TR/T_{1}})}{1-e^{-TR/T_{1}}cos\alpha_{i}}\#\left( 5 \right) \end{aligned}$$

where $\alpha_{i}$ is the flip angle of the ith measurement. If there are at least two measurements with different flip angles, T1 mapping is possible using Equation (5).

qDESS is a 3D T2 mapping method based on the DESS sequence, a well-known, undistorted SNR-efficient 3D imaging technique [30]. In DESS, two contrast images, free induction decay (FID) signals, $S_{fid}$, and ECHO signals, $S_{echo}$, are obtained simultaneously per TR. T2 mapping is performed by utilizing the relationship between the FID signal and ECHO signal ratio as follows [31]:

$$\begin{aligned} \frac{S_{echo}}{S_{fid}}=e^{-2(TR-TE)/T_{2}}\#\left( 6 \right) \end{aligned}$$

In a study by Sveinsson et al. [32], T1 and diffusion effects were considered, particularly when focusing on the knee.

$$\begin{aligned} \frac{S_{echo}}{S_{fid}}=e^{-2(TR-TE)/T_{2}-\left( TR-\frac{\tau}{3} \right)\Delta k^{2}D}{sin}^{2} (\frac{\alpha}{2})(\frac{1+e^{-TR/T_{1}-TR\Delta k^{2}D}}{1-cos\alpha e^{-TR/T_{1}-TR\Delta k^{2}D}})\#\left( 7 \right) \end{aligned}$$

where TR and TE represent the repetition and echo time, respectively. $\alpha$ is the flip angle, D is the diffusivity, and the dephasing per unit length induced by the unbalanced gradient is denoted by $\Delta k =\gamma G\tau$, where G and τ are the spoiler amplitude and duration, respectively. In this study, following Sveinsson et al.[32], we focused on knee imaging and assumed T1 to be 1000 ms, with other sequence parameters based on the SKM-TEA dataset [33].

**Training strategy of deep learning MRI reconstruction**

In this section, the DL training method used in this study is described. In recent years, research on learning methods has been actively conducted with the development of DL networks. The datasets required differed depending on the learning method, and some datasets did not require a ground truth (GT). The representative learning methods include SL, SSL, and unsupervised learning (UL). In SL, a pair of model inputs and reference data are provided, and the error between the model output and reference data is evaluated using a loss function. The weights were updated to minimize the loss. In contrast, SSL assumes a scenario in which a GT is unavailable. In SSL, a pretext task, which is a task related to the original goal, is used for learning. Specifically, the synthetic GT generated from the input data is used as the teacher data, and by learning the transformation to this synthetic teacher data, the model learns the ability to transform the input data to the original GT. UL also assumes a situation in which the GT is unavailable; however, unlike SSL, it does not involve synthesizing teacher data. In the UL, a loss function that evaluates only the output data is used.

In addition to the learning methods, research has also focused on reducing the number of images required for learning. Methods with more than two learning samples are called few-shot learning, those with only one learning sample are called one-shot learning, and those with zero learning samples are called zero-shot (ZS) learning. The ZS learning targeted in this study uses only test data, meaning that there is no need to prepare the data for learning. Therefore, it is fundamentally impossible in SL frameworks that require GT; therefore, SSL or UL frameworks must be used.

SSDU is an SSL framework for image reconstruction proposed by Yaman et al. in 2020. In the SSL framework, no data are required and only US data can be used for training. In a typical SL framework, learning requires paired data from the US and FS k-space data. The learning process in SL is expressed as follows:

$$\begin{aligned} \tilde{\theta}=\min_{\theta} L\left( {y_{U},E}_{U}f_{dnn}\left( y_{\Omega},E_{\Omega};\theta\right) \right)\#\left( 8 \right) \end{aligned}$$

where $\tilde{\theta}$ is the parameter obtained through learning, L is the loss function, $f_{dnn}\left( y_{\Omega},E_{\Omega};\theta\right)$ is the DL network with $\theta$ as the parameter and $E_{\Omega}$ as the internal encoding operator, and $y_{U}$ is the FS data.

By contrast, in the SSDU framework, learning is performed without using the FS data. In this case, the original US region $\Omega$ is divided into two sampling regions: $\Theta$ and Λ. The learning process in SSL is expressed by the following equation.

$$\begin{aligned} \tilde{\theta}=\min_{\theta} L\left( y_{\Lambda},E_{\Lambda}f_{dnn}\left( y_{\Theta},E_{\Theta};\theta\right) \right)\#\left( 9 \right) \end{aligned}$$

Here, $\Omega=\Theta\cap\Lambda$. The goal of the image reconstruction problem is to reconstruct FS k-space data from US k-space data, which can be rephrased as the problem of completing the unsampled region $\Omega$. In the SSL framework, the ability to properly complete the unsampled region $\Omega$ is evaluated by assessing whether the sampling region $\Lambda$, which is not included in the sampling region $\Theta$ of the input k-space data, can be appropriately reconstructed.

Yaman et al. extended SSDU to a ZS learning application. In ZS learning, because only the test dataset is available, training and validation datasets must be generated from the test dataset. Normally, different data are used for the training and validation datasets. However, they solved this problem using different sampling regions for each dataset. Specifically, the US region $\Omega$ is divided into three sampling regions $\Theta$, $\Lambda$, and $\Gamma$, so that $\Omega=\Theta\cap\Lambda\cap\Gamma$.

[R2-4] The network architecture section, as currently written, is highly technical and assumes a deep familiarity with deep learning concepts and MRI reconstruction. Terms like "unrolled network," "conjugate gradient method," and "residual blocks" are used without sufficient explanation. The section does not explain why the specific network architecture was chosen or how it addresses the challenges of qMRI reconstruction.

Thank you for the insightful comment. To improve clarity for readers less familiar with deep learning and MRI reconstruction, we have added brief explanations of key terms such as “unrolled network” and “residual network” in the revised Materials and Methods section (page 9, line 2 and page 10, line 7). We also clarified the motivation for adopting the unrolled architecture and explained that our choice of ResNet was based on preliminary experiments showing instability with plain CNNs. Regarding the conjugate gradient method, we kept the explanation concise, as it is a standard approach for solving linear inverse problems.

Material and Methods: page 9, line 2

We used the architecture of an unrolled network. Unrolled networks are a class of DL methods that integrate iterative optimization algorithms with neural networks, combining physics-based modeling and data-driven learning. This approach is known as SoTA for image reconstruction [35]. In recent years, its high performance has led to its application in qMRI, and its effectiveness has been reported [17,25,26]. Therefore, in this study, we adopt an unrolled network as the backbone model.

Material and Methods: page 9, line 14

where $R(x)$ is a specific regularization term, and $\lambda$ is a parameter that balances the data-consistency term and the regularization term. If $R(x)$ is an L2 norm or another typical convex regularization, iterative methods such as the CG method or gradient descent are often employed.

Material and Methods: page 10, line 5

As (14) is a linear inverse problem, it can be solved using the conjugate gradient method. The conjugate gradient method, employed by Aggarwal et al was used in this study.

Material and Methods: page 10, line 7

The network architecture is shown in Fig 2. In this study, we employed a denoiser structure based on the residual network (ResNet)[36]. ResNet is a model that introduces residual connections and enables the effective training of deep networks. In our preliminary experiments, using a multilayer CNN without residual connections as a denoiser resulted in unstable training. Therefore, we adopted a ResNet-based architecture similar to that proposed by Yaman et al.

[R2-5] The mention of "Equation (S8)" and "Problem formulation" in the supplementary file (S1 File) is problematic because readers cannot access this information without switching to another document. Key equations and formulations should be included in the main text or explained in detail.

Thank you for the valuable suggestion. We have moved the key equations and formulations previously included in the supplementary file (S1 File) into the main text. Equations (10)–(14), which define the reconstruction problem, denoiser, and data consistency steps, are now presented and explained directly in the Materials and Methods section (page 9, line 9 onward). The revised text is as follows:

Material and Methods: page 9, line 9

The accelerated MR reconstruction problem under multicoil/multicontrast conditions is formulated as follows:

$$\begin{aligned} x_{rec}=\underset{x}{argmin} \left\| y_{\Omega}-E_{\Omega}x \right\|_{2}^{2}+\lambda R(x) ,\#\left( 10 \right) \end{aligned}$$

where $R(x)$ is a specific regularization term, and $\lambda$ is a parameter that balances the data-consistency term and the regularization term. If $R(x)$ is an L2 norm or another typical convex regularization, iterative methods such as the CG method or gradient descent are often employed.

The input to the networks was the coil sensitivity and US multi-contrast k-space data, and the output was the reconstructed multi-contrast k-space data. To construct an unrolled network, similar to that of Aggawal et al. We adopted a DL-based L2 norm for the regularization term in Equation (10) as follows:

$$\begin{aligned} x_{rec}=\underset{x}{argmin} \left\| E_{\Omega}x - y_{\Omega} \right\|_{2}^{2}+\lambda\left\| x - D_{w}(x;\theta) \right\|_{2}^{2}\#\left( 11 \right) \end{aligned}$$

Here, $D_{w}$ is called the denoiser, which is responsible for removing artifacts similar to noise from CS, and θ is the parameter of the convolutional neural network (CNN). Equation (11) can be transformed into a problem of alternating convex optimization between variables $x$ and $z$ by introducing a new intermediate variable $z_{n}$.

$$\begin{aligned} z_{n}=D_{w}(x_{n};\theta)\#\left( 12 \right) \end{aligned}$$

$$\begin{aligned} x_{n+1}=\underset{x}{argmin} \left\| E_{\Omega}x - y_{\Omega} \right\|_{2}^{2}+\lambda\left\| x - z_{n} \right\|_{2}^{2}\#\left( 13 \right) \end{aligned}$$

Equation (12) corresponds to the denoiser, and Equation (13) corresponds to the data consistency (DC) layer, representing the process of alternating optimization of variables $x$ and $z$. Equation (13) can be rewritten using the linear operator $\boldsymbol{Q=}\boldsymbol{E}_{\boldsymbol{\Omega}}^{\boldsymbol{H}}\boldsymbol{E}_{\boldsymbol{\Omega}}+\lambda\boldsymbol{I}$ as follows:

$$\begin{aligned} x_{n+1}=\left( E_{\Omega}^{H}E_{\Omega}+\lambda I \right)^{-1}(E_{\Omega}^{H}y_{\Omega}+\lambda z_{n})\Leftrightarrow x_{n+1}=Q^{-1}(E_{\Omega}^{H}y_{\Omega}+\lambda z_{n})\#\left( 14 \right) \end{aligned}$$

As (14) is a linear inverse problem, it can be solved using the conjugate gradient method. The conjugate gradient method, employed by Aggarwal et al was used in this study.

[R2-6] The manuscript does not always explain why certain methods or parameters were chosen. For example, why were specific acceleration factors (AF4, AF8, AF12) selected? What is the rationale behind the 60%-40% split in SSL?

Thank you for the comment. We have added brief explanations for the choice of acceleration factors and data splits. The 60%-40% split follows Yaman et al.’s settings, and AF4–AF12 were selected to cover common and challenging acceleration levels in qMRI. The revised text is as follows:

Material and Methods: page 11, line 9

According to the original study, subsets $\Theta$ and $\Lambda$ were randomly selected such that $\Omega=\Theta\cap\Lambda$, with a 60% to 40% ratio for the split between $\Theta$ and $\Lambda$similar to Yaman et al, which demonstrated strong performance in their study. In the ZSSSL condition, losses$\mathcal{L}_{train}$ and $\mathcal{L}_{val}$ are formulated as follows:

Material and Methods: page 11, line 14

Subsets $\Theta$, $\Lambda$, $\Gamma$, and $\Xi$ were randomly selected such that $\Omega=\Xi\cap\Gamma$ and $\Xi=\Theta\cap\Lambda$, with an 80% to 20% ratio for the split between $\Xi$ and $\Gamma$, and a 60% to 40% ratio for the split between $\Theta$ and $\Lambda$ similar to Yaman et al. In ZSSSL, an early stopping strategy was employed, in which training was stopped if no improvement in $\mathcal{L}_{val}$ was observed for more than 25 epochs.

Material and Methods: page 12, line 23

The following experiments were conducted using three sequences: 2D T2 mapping/MSME (Experiment 1), 3D T1 mapping/VFA-SPGR (Experiment 2), and 3D T2 mapping/DESS (Experiment 3). These sequences have been commonly used in other qMRI studies [4,16,17,30,32,38].  Each experiment was conducted on the US k-space data under the AF conditions of 4 (AF4), 8 (AF8), and 12 (AF12). In single-contrast image reconstruction, AF4 is typically used, with AF8 considered a more challenging condition [18]. In this study, we also investigated AF12, anticipating further acceleration benefits from the use of multicontrast inputs.

[R2-7] The training does not explain why the specific training strategies were chosen or how they address the challenges of qMRI reconstruction.

Thank you for the comment. We have revised the manuscript to clarify the rationale for choosing SL, SSL, and ZSSSL. In particular, we highlight that GT-free strategies like SSL and ZSSSL are well-suited for qMRI, where fully sampled data are costly to obtain. The revised text is as follows:

Material and Methods: page 10, line 24

Three DL methods were compared: SL, SSL, and ZSSSL. The SSDU framework used in a previous study [37] was extended to the multicontrast problem because its implementation was publicly available and reproducible, and extending it to the multicontrast setting was relatively easy. These GT-free methods are highly useful in applications such as qMRI, where the cost of acquiring FS data is high. Fig 3 shows schematic diagrams of the multicontrast extensions of the SL and SSDU.

[R2-8] The training section mentions that "the lost computation results were recalculated from the cache during backpropagation," but it does not explain how this recalculation is performed or what its computational cost is. Provide more details about the implementation of MEL, such as how the cache is managed and how the recalculation process works.

Thank you for the comment. We have added a brief explanation to clarify the implementation details of MEL and how recomputation is handled. The revised text is as follows:

Material and Methods: page 12, line 2

We applied the MEL[27] strategy and gradient accumulation to conserve GPU memory for qMRI reconstruction. We implemented a gradient checkpoint strategy based on MEL[27]. Kellman et al. used a method in which, in addition to gradient checkpointing, the DC layer was recomputed in reverse to gain memory benefits. However, our observations indicated that this implementation did not yield significant GPU memory benefits, so we did not adopt it. Gradient checkpointing is a technique that conserves GPU memory by periodically saving “checkpoints” instead of storing all intermediate results during the forward pass and discarding intermediate outputs containing gradient information. During the backward pass, the necessary intermediate results are recomputed from the nearest checkpoint in the forward direction. In this study, we saved the intermediate outputs of denoiser z_i_ as checkpoints. This process requires additional computation time to recompute the intermediate outputs of the denoiser during the backward pass. However, the maximum memory consumption is limited to the memory used to store the checkpoints and the memory required to retain the intermediate output of a denoiser computation step. Gradient accumulation saves memory by splitting the minibatch sent to the GPU and accumulating gradients, thus simultaneously reducing the amount of data sent to the GPU. In addition, we treated the 3D datasets as multiple k-space datasets by applying a Fourier transform to the readout direction of the k-space data. By combining these methods, we successfully employed the network in this study despite the high dimensionality of the target data.

[R2-9] The presentation of quantitative results could be improved. Some tables and figures are difficult to interpret, and the text does not always provide clear explanations of the results.

Thank you for your comment. To improve the interpretability of the quantitative results, we replaced Tables 2–4 with visual representations (Figures 7, 9, and 11) for Experiments 1 to 3. In addition, we revised the description of memory consumption in Fig. 12 to more clearly explain the impact of MEL. The revised text is as follows:

Results: page 18, line 21

The memory consumption results in Fig 12(a) show that MEL effectively reduced memory consumption, particularly when the number of layers was large. Fig 12(b) shows that the training speed was almost the same with and without the use of the MEL. The results in Fig 12(a) indicate that MEL significantly reduces memory consumption, particularly as the number of layers, filters, and iterations increases. Without MEL, memory usage increases rapidly with network complexity, whereas MEL effectively suppresses this growth. By contrast, Fig 12(b) demonstrates that the training speed remains nearly unchanged regardless of the MEL usage, suggesting that the memory-efficient strategy does not introduce a significant computational overhead. In this study, the memory consumption was 7723 MB for Experiment 1, 1429 MB for Experiment 2, and 2000 MB for Experiment 3.

[R2-10] Some sentences are long and complex, which can make them harder to follow. simplify the language for broader readability

Thank you for the comment. We have conducted a professional English proofreading by a native speaker to improve clarity and readability. In addition, we revised the Discussion section to remove redundant or repetitive expressions. The revised text is as follows:

Discussion: page 19, line 31

Discussion: page 21, line 9
